# Socioeconomic determinants of birth registration in Ghana

**DOI:** 10.1186/s12914-015-0053-z

**Published:** 2015-06-15

**Authors:** Joshua Amo-Adjei, Samuel Kobina Annim

**Affiliations:** Department of Population and Health, University of Cape Coast, Cape Coast, Ghana; Department of Economics, University of Cape Coast, Cape Coast, Ghana

**Keywords:** Birth registration, Right, Privilege, Ghana

## Abstract

**Background:**

Identity registration is not only a matter of human rights but it also serves as an important instrument for planning about health, education and overall development. This paper examines the chances of a child born in Ghana between 2001 and 2006 obtaining legal status of identity.

**Methods:**

Data for this paper were extracted from the 2006 Ghana Multiple Indicator Cluster Survey (MICS). We used discrete choice modelling in estimating the likelihood of child registration in Ghana.

**Results:**

Mother’s education and household wealth are identified to be positively associated with the likelihood of a child being registered. In the context of structural factors, being a resident in the Eastern region of Ghana and rural areas were found to be risk factors for children not being registered. Besides, children who were resident in households where the head is affiliated to Traditional Religion were found to be at significant risk of being unregistered.

**Conclusion:**

Overall, our findings give an impression of birth registration being a privilege for children whose parents are educated, wealthy and resident in urban communities. Policies meant to increase uptake have to be broad-based, targeting the less privileged particularly with practical interventions such as transport vouchers to registration centres. This may help appropriate meaning to international protocols on birth registration as a human right issue to which Ghana affirms.

## Background

The first point of call between individuals and their states is being registered and counted as part of the state, bestowing on them all rights and privileges that the state provides. Dow [1] described birth registration as the first ticket to citizenship without which an individual does not exist legally and could be denied privileges and rights a nation allows. The Plan International [2] elaborated the rights perspective position in the following:

*A birth certificate is the first official acknowledgement of a child’s existence by the State and is essential if they are to access other rights. Where births remain unregistered, there is an implication that children are not recognised as persons before the law … access to fundamental rights and freedoms may be compromised … existence has never been recorded, there is no guarantee that their disappearance will be either … as they will not be included in statistical information about children, their situation cannot be monitored* (p.2).

The 1948 UN Universal Declaration of Human Rights and subsequent establishment of the World Bank aptly recognised human rights and development as intertwined concepts that are not mutually exclusive [3]. Of the cardinal issues raised in the human rights framework is the right of identity. Article 24(2) of the UN International Covenant on Civil and Political Rights (ICCPR) ingenuously states “every child shall be registered immediately after birth and shall have a name”. Article 6(1) further opines: “every human being has the inherent right to life. This right shall be protected by law and no one shall be arbitrarily deprived of his life”. The Convention on Rights of the Child (CRC), one human right instrument that has received more ratifications than any other rights treaty also recognises the rights of every child to birth registration [4]. Unfortunately, about 51 million children are unregistered on yearly basis and the majority of those unregistered are in developing countries in Africa, Asia and Southern America [5].

Research evidence suggests that where individuals have limited or have not been provided with adequate citizenship right through birth registration and issuance of a birth certificate, the capacity of individuals to fully asset their civic, political, legal and social identities are significantly constrained [4]. For instance, the absence of civic citizenship right has positive association with inability of individuals to claim full fundamental human rights. In the same vein, political participation such as voting cannot be fully ascertained or granted to non-citizens whilst social rights, which validate access to health care, education, pension and poverty reduction benefits can also be compromised [6].

Of the broad framework of Millennium Development Goals, availability of quality of data to monitor progress is cardinal. The goals, which directly affect children are universal primary education and improved child mortality. However, without reliable data on children, it will be difficult for countries to monitor, plan and intervene to achieve the goals [7]. Data on births and deaths is one of the best demographic tools for gathering information for population health monitoring. For instance, William Farr’s league of healthy and unhealthy cities of the United Kingdom was principally based on vital statistics [8]. Without comprehensive vital events registration systems, estimations on important child health indicators such as mortality often rely on surveys, of which the quality in developing countries are sometimes doubtful [9]. These concerns about data quality could be dealt with in the presence of complete or high coverage of events, especially births and deaths. According to Szreter [3], the absence of such relevant data could be detrimental to development planning and this will ultimately have negative repercussions on improving life chances. At best, the desire to improve development through health, education, social security etc. may “remain a political rhetoric of human rights and academic discourse of entitlements” [3].

Apart from the human rights and the monitoring of development indicators arguments for scaling-up identity registration include economic growth. This is because identity registration enhances individuals’ entitlements to property [3, 10]. Furthermore, identity registration system promotes sustainability of comprehensive social security system [3]. Individuals without proper identity status can suffer social exclusion, and they are likely to remain in the throes of perpetual oblivion as far as public health monitoring is concerned [11, 12]. Timely registration of children also improves access to education because in countries such as Malaysia, Tanzania, Togo etc., child registration certificates are part of entry requirements into formal school systems [11].

There are, however, certain socioeconomic factors that may enhance or constrain birth/identity registration. The popular enhancing or constraining factors are distance [13], type of place of residence (rural–urban) [14], cultural, institutional, political and legislative conditions [13].

Despite the fact that birth registration enhances social, economic and political rights, it can be utilised for unscrupulous activities. In the apartheid South Africa, civil registers were used for political surveillance and persecution [15], used by the Nazi regime to track and persecute Jews [16], to restrict civil freedom in communist China and Soviet Russia [12] and the Rwandan genocide [17].

However, the various instances of unscrupulous application of birth/identity registration databases should not form the basis for denying individuals this right [3, 18]. The institutions and the individuals entrusted with such data are required to uphold high standards of ethics. There could also be provisions for stern international sanctions against deceitful application of identity registration records [18].

Our goal in this paper is to examine socioeconomic, spatial and demographic factors, which enhance or constrain birth registration in Ghana. We draw on a nationally representative survey data conducted in 2006 by the Ghana Statistical Service (GSS) in collaboration with United Nations Children’s Fund (UNICEF). While adequate coverage of vital events provides complete national data sets for demographic, health, economic, educational and political analysis, there is a dearth of empirical evidence on the socioeconomic determinants of birth registration in Ghana and other developing countries despite the under-registration of births. This paper aims to make a contribution in this filling this void.

### Events registration in Ghana

Several social, cultural, economic and political factors, at both macro and micro levels, have, diversely, affected high enrolment in identity registration in most part of the developing world, particularly in Asia, Latin America, the Caribbean and Africa. In sub-Saharan Africa, for instance, the population without birth registration certificate is about 65 % [13]. Over the years, some concerted efforts have been made to achieve acceptable up-take of birth registration in Africa, although spearheaded by Non-Governmental Organisations (NGOs) such as Plan International and UNICEF.

In Ghana, vital events registration started as far back as 1888. Initially, it was limited to the registration of deaths, which was also confined to expatriate workers in the colonial government service and mining and other merchandise companies. Later in 1912, the system was expanded to include births. Since then, the processes of birth registration have evolved in synergy with legal framework(s) establishing events registration. Beginning with the Cemeteries Ordinance of 1888, event registration legal framework was amended in 1891. The legal framework was changed to Births, Deaths and Burials Ordinance in 1912 and later amended in 1926 and it was subsequently amended to Registration of Births and Deaths, Act 301 of 1965. The various amendments that the legal frameworks have gone through were all generally intended to improve events registration. The current system of event registration in Ghana is managed under the auspices of Ministry of Local Government and Rural Development. The core mandate of the registry is to provide accurate and reliable information on all births and deaths, which occur within Ghana for socio-economic development of the country through their registration and certification.

At the international front, Ghana, in February 1990, was the first country in Sub-Saharan Africa that ratified the UN Convention on the Rights of the Child. After more than a decade, coverage of birth registration in the country as at 2008 was 51 % and coverage of all vital events (birth, death and marriage) was 25 %. To improve birth registration in the country, the financial cost has been scrapped to encourage registration within the first 12 months after birth. Registration is also limited to a registration centre in the region of delivery. We hypothesize that the low costs associated with birth registration in the country are, therefore, not expected to result in differences in registration by parental wealth index.

## Methods

### Data

The analysis in this paper is based on data extracted from the Multiple Indicator Cluster Surveys (MICS). With support of UNICEF, MICS is intended to complement other existing national data sets especially, the Demographic Health Surveys to provide information on health, education, child protection and HIV/AIDS. Beginning from the mid-1990s, four rounds of the survey have been collected in about 65 countries. In each country, the dataset is nationally representative. The sampling frame was based on the 2005 Ghana Living Standard Survey (GLSS5). The frame was first stratified into the 10 administrative regions in the country, then into urban and rural enumeration areas (EAs). The 2006 MICS employed a two-stage stratified sample design. At the first stage of sampling, 300 census EAs (124 urban and 176 rural) were selected. These are a subsample of the 660 EAs (281 urban and 379 rural) selected for the GLSS5. The clusters in each region were selected using systematic sampling with probability proportional to their size. In three of the regions in the country (Northern, Upper East and Upper West), there was intentional oversampling to derive a representative sample. Data was available on 3466 children and after data cleaning and management, 98 % of the data was used for our analysis. Ghana has participated in the first and third rounds of MICS (1995 and 2006) and these datasets are available on demand. At the time of writing this paper, the 2011 dataset had not become publicly available. However, the 2006 version was available and freely accessible at www.unicef.org. The MICS data covers three thematic areas; household, women and children’s characteristics. Household characteristics data captures education, child labour, water and sanitation, salt iodization, insecticide-treated mosquito nets, and support to children orphaned and made vulnerable by HIV/AIDS, with optional modules for disability, child discipline, security of tenure and durability of housing, source and cost of supplies for ITNs, and maternal mortality. Information about children in the survey includes birth registration and early learning, vitamin A, breastfeeding, care of illness, malaria, diarrhoea immunization, and anthropometry. There are optional modules on child development, and source and cost of supplies of ORS, antibiotics and anti-malarial drugs. The optional modules are targeted at meeting needs of specific country needs. The overall response rate was 95 % [19]. The MICS dataset was preferred because it is more child-centred and it also contains information on the exact question of whether the child is registered or not.

### Methods of data analysis

The first part of the analysis is based on univariate (it examines patterns of self-reported reasons for non-registration) and bivariate (it explores relationship between proportion children registered by socioeconomic covariates). Basic descriptive statistics including chi-square are used to test whether there is significant difference between responses of birth registration and its correlates. The respondents, whose children were not registered, were asked to indicate their reasons for non-registration of their births. The responses are depicted graphically. We further employed logistic regression technique (discrete choice model) to provide a response to examine variables, which are associated with registration of a child at birth. The use of discrete choice model is informed by the nature of our dependent variable, which was measured as a binary factor. Explanatory variables used in the logistic model are: the child’s age and its square, wealth of household, mother’s education, religion of head of household and rural–urban residence. Model 1 varies from the others based on the inclusion of the vaccination variable. Models 2 – 5 vary based on the way mother’s age is treated. Model 6 includes place of delivery. We further explored the square of child’s age to examine the non-linearity, which was observed from the bivariate analysis. Both the descriptive and the inferential analyses were weighted to take care of over and under sampling associated with nationally representative surveys. The MICS data provides a weighting variable, which helps to offset over- and under-sampling in some regions. The weighting factors, thus, help to generate generalizable results for the entire country.

## Results

Table 1 presents proportions of children between the ages 0–59 months who are not registered by individual, household and community variables. Corresponding levels of association between the independent variables and the dependent variable are also indicated based on the chi-square test. Apart from sex of the child, which showed no significant association with likelihood of being registered, all the other control variables showed significant association with child registration. A majority (56.89 %) of children in rural areas had not been registered. At another spatial level, thus, in terms of regional distribution, a little over two-thirds of children (61 %) in the Eastern region had not been registered. Unsurprisingly, Greater Accra, which hosts the national capital, reported the least proportion of children (27 %) who had not been registered. For the remaining regions, identity registration ranged roughly from 43 % in the Ashanti to 52 % in the Northern region. As expected, child registration practices improved with increasing maternal education (Table 1).

**Table 1:** Proportion of children 0–59 months who do not have birth registration records

Correlates	Per cent without birth registration	Number without birth registration
Child’s Sex		
Male	46.99	840
Female	48.25	808
Child’s Age		
0 - 11 months	55.47	405
12 - 23 months	39.60	281
24 - 35 months	41.64	277
36 -47 months	48.99	351
48 - 59 months	52.09	335
Settlement		
Urban	30.86	382
Rural	56.89	1267
Region		
Western	51.42	178
Central	47.06	142
Greater Accra	27.41	123
Volta	51.33	134
Eastern	60.87	282
Ashanti	42.66	214
Brong-Ahafo	49.87	155
Northern	52.27	303
Upper East	45.13	66
Upper West	49.34	52
Mother’s Education		
None	57.50	772
Primary	50.57	381
Middle/JSS	39.76	444
Secondary+	20.57	52
Wealth Index Quintiles		
Poorest	68.86	539
Second	59.05	489
Middle	41.97	287
Fourth	36.96	230
Richest	18.87	103
Religion		
No Religion	64.05	199
Christian	44.19	905
Moslem	37.67	231
Traditional	70.59	226
Spiritualist and Other	51.09	88
Total	47.60	1,649

Children, whose parents have had higher formal education (79.43 %), were more likely to be registered than those whose parents had comparatively attained lower education. Similar patterns of increased chances of children being registered were observed with higher household wealth. For instance, whereas about 69 % of children from poorest households had not been registered, less than one-fifth (18.87 %) from wealthiest households was not registered.

The results also point to widespread variations in child registration by religious affiliation of the head of the household. Approximately, 71 % of children whose heads of household were affiliated to Traditional Religion were not registered. Children who come from households with Moslems as the heads were less likely to be unregistered.

**Fig. 1:**
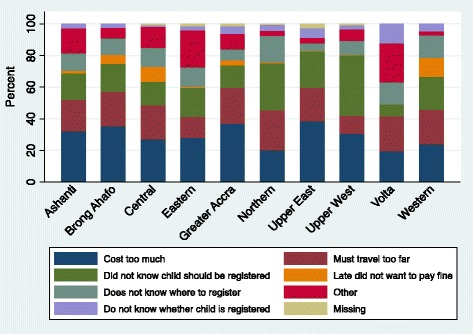
Reasons for child non-registration in Ghana

Turning to spatial differences, the cost of registration was the commonest reason given by parents for not registering their children in all the regions (Fig. 1)

**Fig. 2:**
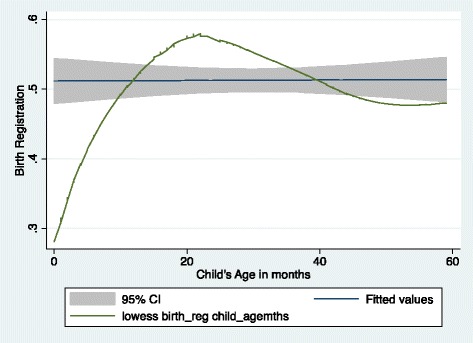
Bivariate relationship between child’s birth registration and age in Ghana

To determine the variables that statistically influence identity registration, several equations were estimated and the results are presented in (Table 2). Model 1 suggests that age of a child is associated with the probability of registering the child at birth. Thus, older children are about 7 % likely to be registered. The probability remains stable at around 8 % in Models 2–5 and rises steadily to 11 % in Model 6. However, as evidenced in Fig. 2, the relationship between the probability of birth registration and a child’s age is non-linear. As shown in Fig. 2, at lower ages (in months) there is a higher likelihood of the child being registered but this diminishes as the child approaches 60 months. Figure 2 further indicates that the turning point where the child is less likely to be registered is about 24 months. Also, from Fig. 1, there is an indication that children are more likely to be registered just about the time they are turning 5 years old, perhaps to prepare them for formal schooling, which begins at age 6.

**Table 2:** Binary logistic regression of the likelihood of a child born in Ghana being registered

Explanatory factors	Model 1		Model 2		Model 3		Model 4		Model 5		Model 6	
	OR	95 % CI	OR	95 % CI	OR	95 % CI	OR	95 % CI	OR	95 % CI	OR	95 % CI
Child age-months	1.07**	[1.05,1.10]	1.08**	[1.06,1.11]	1.08**	[1.06,1.11]	1.08**	[1.06,1.11]	1.08**	[1.06,1.11]	1.11**	[1.07,1.14]
Child age squared	1.00**	[1.00,1.00]	1.00**	[1.00,1.00]	1.00**	[1.00,1.00]	1.00**	[1.00,1.00]	1.00**	[1.00,1.00]	1.00**	[1.00,1.00]
Maternal education^a^												
Primary	1.57**	[1.15,2.14]	1.58**	[1.16,2.16]	1.56**	[1.15,2.12]	1.61**	[1.19,2.20]	1.60**	[1.17,2.18]	1.54*	[1.05,2.27]
Middle/JSS	1.80**	[1.30,2.48]	1.82**	[1.32,2.52]	1.88**	[1.37,2.60]	1.88**	[1.36,2.59]	1.84**	[1.33,2.54]	2.06**	[1.37,3.12]
Secondary plus	1.70^+^	[0.92,3.12]	1.72^+^	[0.94,3.17]	1.80^+^	[0.97,3.34]	1.74^+^	[0.94,3.21]	1.72^+^	[0.94,3.18]	1.13	[0.53,2.43]
Paternal education^a^												
Primary	1.37^+^	[0.97,1.94]	1.41^+^	[1.00,1.99]	1.42*	[1.01,2.01]	1.42*	[1.00,2.00]	1.40^+^	[0.99,1.98]	1.34	[0.89,2.03]
Middle/JSS	1.66**	[1.20,2.28]	1.71**	[1.24,2.34]	1.66**	[1.21,2.28]	1.70**	[1.24,2.34]	1.70**	[1.23,2.33]	1.55*	[1.04,2.30]
Secondary plus	1.61*	[1.04,2.48]	1.69*	[1.10,2.60]	1.64*	[1.07,2.53]	1.68*	[1.09,2.59]	1.68*	[1.09,2.59]	1.94*	[1.14,3.30]
Wealth status^b^												
Below average	1.48**	[1.11,1.96]	1.54**	[1.16,2.04]	1.54**	[1.16,2.04]	1.53**	[1.15,2.03]	1.53**	[1.15,2.03]	1.42*	[1.01,2.00]
Average	3.49**	[2.41,5.06]	3.62**	[2.50,5.24]	3.76**	[2.59,5.45]	3.68**	[2.54,5.32]	3.61**	[2.50,5.22]	3.23**	[2.06,5.09]
Above average	3.00**	[1.99,4.53]	3.12**	[2.07,4.71]	3.22**	[2.13,4.85]	3.17**	[2.10,4.79]	3.11**	[2.06,4.70]	2.98**	[1.78,5.00]
Highest	7.87**	[4.53,13.67]	8.17**	[4.70,14.21]	8.19**	[4.70,14.26]	8.13**	[4.67,14.16]	8.13**	[4.68,14.13]	5.39**	[2.68,10.84]
Type of residence												
Urban	1.38*	[1.02,1.87]	1.41*	[1.04,1.91]	1.40*	[1.04,1.90]	1.38*	[1.02,1.87]	1.40*	[1.03,1.89]	1.34	[0.91,1.97]
Child sex			0.97	[0.79,1.19]	0.97	[0.79,1.19]	0.97	[0.79,1.19]	0.97	[0.79,1.19]	1.10	[0.85,1.42]
*Region* ^c^	0.96	[0.78,1.18]										
Western			1.50	[0.92,2.45]	1.48	[0.90,2.44]	1.46	[0.89,2.39]	1.47	[0.90,2.41]	1.60	[0.87,2.94]
Central	1.48	[0.90,2.42]	1.82*	[1.10,3.02]	1.85*	[1.11,3.07]	1.84*	[1.11,3.05]	1.82*	[1.10,3.01]	2.15*	[1.11,4.18]
Greater Accra	1.77*	[1.07,2.93]	1.45	[0.83,2.54]	1.48	[0.84,2.62]	1.46	[0.84,2.56]	1.45	[0.83,2.53]	1.55	[0.77,3.11]
Volta	1.45	[0.83,2.54]	2.07**	[1.23,3.48]	2.15**	[1.28,3.64]	2.07**	[1.23,3.49]	2.06**	[1.23,3.46]	2.51**	[1.30,4.85]
Eastern	2.16**	[1.28,3.65]	1.00	[1.00,1.00]	1.00	[1.00,1.00]	1.00	[1.00,1.00]	1.00	[1.00,1.00]	1.00	[1.00,1.00]
Ashanti	1.00	[1.00,1.00]	1.61*	[1.01,2.56]	1.62*	[1.01,2.59]	1.59^+^	[0.99,2.54]	1.59^+^	[1.00,2.54]	1.95*	[1.10,3.48]
Brong-Ahafo	1.58^+^	[0.99,2.52]	1.74*	[1.06,2.86]	1.84*	[1.11,3.04]	1.74*	[1.06,2.85]	1.74*	[1.06,2.86]	2.25*	[1.18,4.29]
Northern	1.72*	[1.05,2.83]	4.16**	[2.55,6.79]	4.18**	[2.55,6.86]	4.23**	[2.59,6.92]	4.15**	[2.54,6.78]	5.30**	[2.91,9.66]
Upper East	4.12**	[2.52,6.74]	7.97**	[4.69,13.54]	8.39**	[4.89,14.38]	8.06**	[4.73,13.73]	8.01**	[4.71,13.62]	11.31**	[5.78,22.11]
Upper West	7.70**	[4.52,13.09]	4.94**	[2.90,8.39]	5.16**	[3.00,8.85]	4.94**	[2.90,8.42]	4.88**	[2.87,8.31]	5.25**	[2.75,10.06]
Religion of head of household^d^												
Christian	1.15	[0.78,1.69]	1.15	[0.78,1.70]	1.17	[0.79,1.72]	1.16	[0.79,1.71]	1.15	[0.78,1.70]	0.84	[0.52,1.36]
Moslem	1.95**	[1.28,2.97]	2.00**	[1.32,3.04]	2.00**	[1.31,3.04]	1.99**	[1.31,3.03]	2.00**	[1.32,3.04]	1.49	[0.89,2.48]
Traditional	0.70	[0.43,1.15]	0.73	[0.45,1.18]	0.72	[0.44,1.17]	0.73	[0.45,1.19]	0.74	[0.45,1.19]	0.57+	[0.31,1.06]
Spiritualist	1.74^+^	[0.92,3.28]	1.75^+^	[0.93,3.28]	1.79^+^	[0.97,3.29]	1.78^+^	[0.95,3.32]	1.76^+^	[0.94,3.28]	1.36	[0.64,2.88]
Vaccination												
*Marital status* ^e^	5.64**	[2.18,14.58]										
Formerly married			0.09	[0.00,2.18]	0.09	[0.00,2.02]	0.09	[0.00,2.11]	0.09	[0.00,2.11]	1.00	[1.00,1.00]
Never married	0.09	[0.00,2.16]	1.00	[1.00,1.00]	1.00	[1.00,1.00]	1.00	[1.00,1.00]	1.00	[1.00,1.00]	1.00	[1.00,1.00]
Mother’s age (single years)^f^	1.00	[1.00,1.00]	1.01	[0.99,1.02]					1.07	[0.94,1.23]		
Age cohort												
20-24					1.69	[0.57,4.99]						
25-29					2.68+	[0.92,7.81]						
30-34					1.75	[0.60,5.10]						
35-39					2.45	[0.83,7.17]						
40-44					1.61	[0.53,4.87]						
45-49					2.76+	[0.84,9.08]						
Adolescent							0.74*	[0.55,0.98]	1.0	[1,00,1.00]	0.80	[0.57,1.13]
Mother’s age^2^									1.00	[1.00,1.00]		
Place of delivery^g^												
Public health centre												1.73**
Private health centre												1.31
N	2450		2450		2450		2450		2450		1591	
Log likelihood	−1327.63		−1337.19		−1326.06		−1334.39		−1336.63		−855.25	
Hosmer-Lemeshow	22.80(0.00)		19.94(0.01)		13.22(0.07)		13.03(0.07)		12.82(0.08)		9.73(0.20)	

Base categories: Mother’s and Father’s education (No Education)^a^; Wealth (Lowest)^b^; Region (Eastern)^c^; Religion (No religion)^d^; Marital Status (Currently Married)^e^; Mother’s Age category (15–19)^f^; Place of delivery (Home)^f^

^+^p < .10, *p < .05, **p < .01

Mothers with some formal primary, secondary and other higher educational qualification were more likely to register their children than mothers with no education. The relationship between maternal education and birth registration is significant in all but Model 6, which includes place of delivery (public versus private health centre). In a similar respect, wealthier households were more likely to register their children than poorer households (Table 2) compared to children resident in poor households, regardless of the control factors. Rural dwellers were less likely to register their children than urban dwellers. With no religion as base group, children from Moslem households were at a fairly constant and significant higher likelihood of being registered in all the estimation models shown in Table 2.

## Discussion

This study explored identity registration in Ghana with emphasis on factors that enhance the chances of a child gaining identity at birth. Overall, close to half (47.6 %) of Ghanaian children born between 2001 and 2006 had not been registered. The cost of registration of children and lack of knowledge about the need for it dominated the reasons for not registering a child at birth. The discrete choice modelling analysis shows that children had higher chances of staying unregistered if their parents and heads of households were affiliated to Traditional Religion, resident in rural communities, in the Eastern Region, were poorer and less educated.

Our finding that maternal education improves the likelihood of children being registered is not surprising as it is consistent with normative and empirical evidence. Castro and Rud [14] found from Peru and Costa Rica similar issues relative to child registration and maternal education. Among the many returns to education is an expectation that it will eventually increase the stock of quality and quantity of available information. This finding is, therefore, consistent with our expectations. Like in all countries, evidence of birth registration is required before one could process passport application in Ghana and well-educated women are more likely to be cognisant of this practice than those who are not educated. Female formal education is a tool for poverty reduction. In this study, we have demonstrated that the financial cost associated with child registration was a barrier to registration. Formal education for women therefore provides a useful strategy for improving child registration.

We also noted that child registration peaked at the turning point (age approximately 20 months) where penalties are applied before it tappers off after the 20^th^ month after delivery. This makes economic sense, as people will often desire to maximise benefits but minimise costs. In the light of this observation, removing penalties associated with late registration may help to improve birth registration. Our proposal emanates from the fact that birth registration is a right. As a result, financial and institutional barriers should not determine who does or doesn’t get registered.

We recommend that as the country seeks to improve identity registration by minimizing the financial burden associated with birth registration, other indirect costs imposed through spatial or physical accessibility requires attention. Presently, the restriction of registration to regions, where children were born is not likely to propel the country to universal child registration. Regardless of the reason for delivery in a region other than the applicant’s usual place of residence, registration would normally not be allowed. Applicants for birth registration are ‘required’ to do so in the region where delivery occurred. This regulation seems to counterproductive as noted from the Figure: some respondents mentioned the role of distance between their localities and registration centres. One of the possible measures that can help remove barriers occasioned by distance is making the process more flexible, without insisting on registering children in the specific regions in which they were born since the cost of travelling can become a disincentive to registration. Another strategy is to transform the registration process from manual to digital. In this way, no matter where an individual registers his/her child, the details could be connected to the regional as well national databases.

The empirical model also revealed significant spatial variations in child registration in the country. Children born in the three northern regions (Northern, Upper East and Upper West) had better chances of being registered. Although it is difficult to tease out specific factors contributing to this observation, it seems that the Community Health Services Programme (CHPS) [20], which has operated in the northern regions longer than in other areas might be contributing to improvements in child registration. CHPS seeks to promote maternal and child health particularly targeting people in deprived communities. Registration of vital events, including births forms part of the activities of CHPS personnel and this could have possibly contributed the observed spatial patterns.

In spite of the relevant findings from this study and the useful suggestions, by using cross-sectional data, we are constrained to make causal claims. Cross-sectional data may be affected by re-call bias, particularly among a sample that has high illiteracy rates. Consequently, we cannot impute causation into the findings.

## Conclusions

Birth registration and subsequent issuance of certificate does not only promote human rights to citizenship but also facilitates human rights to good health, education, social security and overall human development. Therefore, timely registration of children should be pursued as a right issue. However, findings from this study seem to suggest that it is more of a privilege for a little over half of children whose parents are educated, wealthy and live in urban areas. Yet, without a comprehensive birth, measures aimed at monitoring progress of achieving the MDGs will continue to rely on surveys, which may not be as comprehensive as continuous birth registration system. Policies and programmes meant to increase uptake have to be broad-based, targeting the less privileged (those in rural areas, uneducated mothers, poor households etc.). This may help give proper meaning to country’s signatory to international protocols on birth registration as human rights.
